# Bone-Healing Enhancement Using Particulate Biomaterials and Fibrin-Based Compounds: A Narrative Literature Review of Evidence in Animal Models

**DOI:** 10.3390/ma19020224

**Published:** 2026-01-06

**Authors:** Lívia Maluf Menegazzo Bueno, Camila Pascoal Correia dos Santos, Paola Tatiana Espinosa Cruel, Gabriela Romanini, Lithiene Ribeiro Castilho Padula, Cindel Regina dos Santos Oliveira, Daniela Vieira Buchaim, Rogerio Leone Buchaim

**Affiliations:** 1 Medical and Dentistry School, University Center of Adamantina (FAI), Adamantina 17800-000, Brazil; likamaluf@usp.br (L.M.M.B.); gabrielaromanini@fai.com.br (G.R.); lithiene@fai.com.br (L.R.C.P.); danibuchaim@alumni.usp.br (D.V.B.); 2 Department of Biological Sciences, Bauru School of Dentistry (FOB-USP), University of São Paulo, Bauru 17012-901, Brazil; camila.pcs@usp.br (C.P.C.d.S.); paolaespinosacruel@usp.br (P.T.E.C.); 3 Postgraduate Program in Science, Children’s Oral Health, Araçatuba School of Dentistry (FOA-UNESP), São Paulo State University, Araçatuba 16015-050, Brazil; cindel.oliveira@unesp.br; 4 Graduate Program in Anatomy of Domestic and Wild Animals, School of Veterinary Medicine and Animal Sciences, University of São Paulo (FMVZ-USP), São Paulo 05508-270, Brazil; 5 Department of Postgraduate, Dentistry School, Faculty of the Midwest Paulista (FACOP), Piratininga 17499-010, Brazil

**Keywords:** biomaterials, particulate matter, bone substitutes, fibrin tissue adhesive, hydroxyapatites, bone regeneration, scaffolds, tissue engineering

## Abstract

The human body’s ability to recover from bone injuries is remarkable; however, in specific conditions, interventions are required to restore function and prevent complications. To accelerate osteogenesis, several strategies have been explored, including grafts, biomaterials, and adjuvant therapies. The aim of this narrative review was to analyze the preclinical evidence regarding the combination of particulate biomaterials and fibrin derivatives for bone regeneration. Publications using hydroxyapatite, bovine bone, β-tricalcium phosphate, and bioglass in association with fibrin glue, heterologous fibrin sealants, or platelet-rich fibrin were examined to identify recurrent experimental patterns and biological outcomes. According to the studies, hydroxyapatite and bovine bone were the most frequently investigated scaffolds, whereas fibrin glue and heterologous fibrin sealants showed consistent adhesion and favorable host response profiles in animal models. β-tricalcium phosphate demonstrated faster remodeling but lower volumetric stability, and bioglass showed high bioactivity in isolated reports. Despite heterogeneity in particle size, fibrin formulations, defect models, and follow-up periods, most studies reported enhanced bone deposition, vascularization, and integration when particulate biomaterials were combined with fibrin-based matrices. Overall, the evidence suggests that these combinations promote more organized and biologically favorable bone healing under experimental conditions. Future translational and clinical research is required to standardize protocols and determine the therapeutic applicability of these strategies in human bone repair.

## 1. Introduction

**Figure 1:**
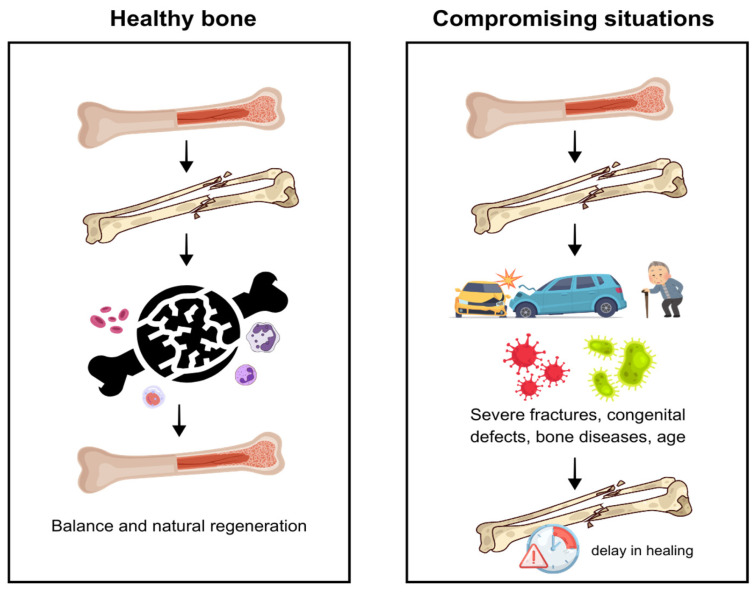
Bone healing in healthy and compromising conditions. Healthy bones show natural regeneration, while severe fractures, disease, or age may delay healing.

Bone tissue plays essential roles in support, protection, and structural integrity, relying on specialized cells and a mineralized extracellular matrix rich in type I collagen and hydroxyapatite [1,2,3]. Although it exhibits remarkable regenerative capacity, severe injuries, extensive fractures, congenital defects, or systemic conditions may compromise healing and require therapeutic intervention to restore function and prevent complications [4,5] (Figure 1). For this reason, several strategies—such as grafts, biomaterials, and physical therapies—have been explored to enhance osteogenesis [6,7,8,9].

Autogenous grafts are effective for repairing bone defects and widely used in orthopedic, dental, and maxillofacial procedures, but limitations such as donor-site morbidity and the need for additional surgery encourage the search for alternatives [10,11,12,13]. Biomaterials, natural or synthetic, interact compatibly with tissues, and in particulate form they fill bone defects, adapt to the surgical site, and support regeneration [14,15]. Advances in materials science have enabled the development of synthetic substitutes capable of mimicking bone or releasing bioactive substances [16].

Common particulate biomaterials include hydroxyapatite, tricalcium phosphate, xenogeneic or allogeneic grafts, biodegradable ceramics, and synthetic substitutes, selected according to the defect and clinical needs [17,18,19]. These materials may also be combined with fibrin derivatives to improve their stability and adaptation to the surgical site.

Among the materials frequently used in combination with these particulates, fibrin derivatives play a key role in enhancing cohesion and biological integration. Fibrin glue is a surgical adhesive formed during the coagulation process and elicits an appropriate host response in its specific application, natural degradation, and no need for removal [20,21,22].

To synthesize the concepts introduced above, particulate biomaterials and fibrin-based matrices represent complementary strategies in bone regeneration: particulates provide structural support and fill complex defects, while fibrin derivatives offer biological adhesion and early matrix stabilization. Despite being frequently combined in experimental studies, the biological rationale and comparative performance of these associations have not yet been reviewed in an integrated way. Therefore, a focused narrative review is relevant to bring together these findings, clarify recurring experimental patterns, and highlight their potential translational implications.

Considering these aspects, this work was structured as a narrative literature review, allowing a qualitative and integrative synthesis of preclinical findings from animal studies. The objective of this review is to examine and compare experimental approaches for repairing bone defects using particulate biomaterials and fibrin derivatives in animal models. Although these studies do not directly translate into clinical outcomes, they provide fundamental biological evidence that supports future translational research and the potential development of clinically applicable strategies.

## 2. Particulate Biomaterials

The concept of *biomaterial* has been refined over time. Earlier definitions focused mainly on inert materials placed in the body, but the scope of the field has expanded significantly. According to the most recent international consensus, a biomaterial is now understood as a material intentionally designed to interact with biological systems in a way that guides or supports diagnostic or therapeutic procedures. This updated perspective provides a clearer framework for discussing particulate biomaterials in bone regeneration [23].

Rather than reiterating basic properties already described earlier, the subsections below emphasize the key differences among particulate biomaterials, which help explain the variability in regenerative outcomes observed across preclinical studies.

In the context of bone regeneration, particulate biomaterials represent one of the most widely adopted strategies in preclinical research due to their versatility, ease of handling, and ability to adapt to irregular defect geometries. Their physicochemical characteristics—including porosity, crystallinity, particle size, and degradation rate—directly influence biological performance in vivo, affecting angiogenesis, osteo-conduction, cellular infiltration, and mechanical stability within bone defects. Although these materials are studied in animal models, this preclinical evidence provides essential mechanistic insight that supports future translational applications in human bone regeneration [24,25,26].

Materials for bone structure replacement and regeneration are classified as biomaterials. A biomaterial is any substance, natural or synthetic, other than drugs or pharmaceuticals, that can be used for extended periods to treat, replace, or improve tissues, organs, or body functions.

These biomaterials can come in various forms, such as blocks, granules, membranes, and microspheres, or with varying compositions. The format is chosen based on the type of bone defect: height or thickness reconstructions require block materials, while particles are suitable for filling existing or surgically created defects [24,25]. Used in the reconstruction of bone defects, they provide support for angiogenesis and bone neoformation. In general, these materials have good compressive strength and low tensile strength, similar to human bone [26].

### 2.1. Bioceramics

Ceramics, or bioceramics, are the most common synthetic materials used in bone repair. They can be presented in the form of powder, coatings, or prostheses and are used to repair, augment, or replace diseased tissues such as bones, joints, and teeth [27,28]. Calcium phosphate (Ca/P)-based nanostructured bioceramics is an innovative class of biomaterials with characteristics that can differ substantially from conventional formulations. These materials may exhibit bioactivity, high surface area derived from nanoscale grains and microporosity, and, in some cases, a crystallographic profile that approximates that of natural bone apatite, contributing to their biocompatibility [29,30].

These bioceramics with interconnected micro- and nanostructures provide significant advantages, such as improved cell adhesion and proliferation, due to their highly specific surface, micro- and nanoscale porosity, and favorable topography. These structural properties confer greater bioactivity, solubility, and biodegradability, especially when applied in vivo or in simulated environments [31,32,33]. In addition, the presence of nanometric grains and an interconnected microporous microstructure creates ideal conditions for wettability, capillarity, and cell adhesion, promoting osteo-induction, osseointegration, and the formation of new bone tissue [34,35].

The particle size of the biomaterial directly impacts the surface area available for interaction with cells and biological fluids; larger particles can result in prolonged absorption time [36]. Precise control of macro- and microporosity is crucial for the effectiveness of the grafted material. Cell colonization of bone substitutes depends on porosity characteristics, such as pore size and distribution, as well as the number and size of interconnections between macropores. These interconnections form a network of tunnels that facilitates the passage of biological fluids, allowing bone cells to enter and promoting new bone formation [37].

Moreover, preclinical models consistently demonstrate that the balance between porosity and mechanical integrity is critical: excessive porosity may compromise mechanical stability, whereas insufficient porosity limits vascular ingrowth and cell migration. These observations highlight the importance of optimizing pore architecture based on the biological demands of each defect [36,37].

After implantation of a bioceramic material, new bone gradually replaces the graft as it is absorbed. Therefore, the periods of graft absorption and bone regeneration must be synchronized to ensure effective bone regeneration [38]. Absorption is a desirable feature in certain types of implants, where the degradation process occurs simultaneously with bone formation in replacement [39].

Therefore, the synchronization between material resorption and new bone formation, repeatedly observed in vivo, remains one of the key determinants of successful regenerative outcomes in animal models and a major consideration for the development of clinically relevant biomaterials [38,39].

In animal studies, these structural features have been shown to accelerate early cell recruitment, enhance osteoconductive behavior, and facilitate progressive mineral deposition within bone defects, demonstrating the importance of micro- and nanoscale topography for preclinical performance [31,32,33,34,35].

### 2.2. Hydroxyapatite

Hydroxyapatite (HA) is a calcium phosphate widely used as a substitute in bone grafts due to its biomimetic similarity to the inorganic components of bones and teeth [40]. Comprising 30 to 70% of the mass of bones and teeth, HA is the main mineral in these structures [41].

For decades, HA was the only bioceramic in the calcium phosphate system used in bone regeneration and replacement due to its similarity to the mineral phase of these tissues [42]. However, despite its favorable biocompatibility, defined according to the 2018 Chengdu consensus as “the ability of a material to perform with an appropriate host response in a specific application” [23], its intrinsic brittleness, low mechanical strength, and slow degradation limit its applicability in defects requiring mechanical support or faster remodeling [43]. Importantly, as emphasized by Williams [44], no material is intrinsically biocompatible; biocompatibility is a context-dependent property determined by the specific tissue, application, and intended function. Given these limitations, other calcium phosphates, such as tricalcium phosphate and amorphous calcium phosphate, have gained prominence as alternative biomaterials [42,45].

In vivo studies reinforce that HA’s slow biodegradation allows it to act as a long-term structural scaffold, supporting gradual bone formation while maintaining defect volume. However, this same property limits its application in defects requiring rapid remodeling, which is why alternative calcium phosphates have gained relevance in recent preclinical investigations.

### 2.3. β-Tricalcium Phosphate (β-TCP)

β-Tricalcium phosphate (TCP-β) was one of the first calcium phosphate compounds used as a bone substitute, standing out for its osteo-integrative and osteoconductive properties [46,47]. Compared to hydroxyapatite (HA), TCP-β has a higher solubility rate, which favors the absorption of the biomaterial in contact with body fluids and positively impacts osteo-induction, osseointegration, and bone repair [48,49].

Biphasic bioceramics composed of HA and TCP-β have been developed, which show faster absorption than pure calcium phosphates due to increased solubility [50]. The high solubility of these apatite phases confers superior physicochemical and biological properties compared to HA, making these materials more suitable for applications requiring rapid bone formation [48].

Preclinical research further demonstrates that the higher solubility of β–TCP enhances ionic release, which stimulates early vascularization and promotes a more dynamic remodeling response compared to HA. This feature makes β–TCP particularly advantageous in critical-sized defects where rapid turnover is required [46,47,48,49,50].

Additionally, biphasic compositions allow fine control of degradation kinetics, as confirmed in animal studies, enabling a balance between mechanical stability (provided by HA) and biological activity (enhanced by β–TCP) [48,49,50,51,52].

The combination of TCP with HA has proven to be an effective alternative for accelerating HA absorption, with the dissolution rate of the mixture being controlled by the amount of TCP used [50]. Due to its biodegradable nature, TCP in alpha and beta forms has attracted interest in the field of biomaterials, being used in orthopedics and dentistry to fill cavities and bone defects, as well as assisting in the fixation of soft tissues [46]. Among the polymorphic phases of TCP, the β phase is especially relevant due to its chemical stability and adequate absorption speed for applications in bone implants [48,52].

### 2.4. Bioglass

Glasses, among bioceramics, are widely recognized for their high activity and effectiveness in bone regeneration. When implanted, they interact with interstitial fluid, triggering physical–chemical and cellular reactions that result in the formation of a layer of biological hydroxyapatite and mineralized osteoid on their surface. This process promotes bone adhesion, creating a cohesive and resistant interface [53]. The ability of a bioglass to bond to bone tissue, degrade biologically, and form this apatite layer varies according to its composition and the ratio of its components [54]. Animal studies consistently report accelerated osteoid formation around bioglass particles, underscoring their high surface reactivity and ability to rapidly establish a bioactive interface with surrounding tissues [53,54].

At bone sites, bioglasses adhere to collagen, growth factors, and fibrin, creating a porous matrix that facilitates the infiltration of osteogenic cells. Although this matrix provides compressive support, it does not provide structural support [55,56].

The biological properties, including favorable host response, osteo-induction, bioactivity, and, in some cases, the ability to stimulate angiogenesis make bioglasses highly desirable. Several formulations have been developed, ranging from highly reactive ones that bind to soft tissues to those that prioritize mechanical properties or biodegradation. These materials can be used in blocks, granules, as components of composite materials, or as support for cell adhesion, proliferation, and differentiation [55]. The addition of bioglass to hydroxyapatite aims to improve its properties, increasing chemical dissolution and enhancing its bioactivity [57].

Silica-based biomaterials are known for their beneficial effects on bone healing. Although the exact mechanism is not fully understood, bioactive glasses not only provide calcium and phosphate ions for HA formation but also promote osteoblast proliferation and differentiation [58,59]. The versatility of bioactive glasses stems from their chemical composition, which adapts well to the clinical application site without causing damage to surrounding tissues, making them well tolerated by surrounding tissues in the context evaluated [60]. Silica, an inorganic polymer, contains siloxane groups (Si-O-Si) internally and silanols on its surface. The high density of silanol groups (Si-OH) on the surface of amorphous silica is crucial for HA growth and bone formation in vitroceramics [61].

The capacity of bioglass to modulate both early inflammatory responses and later osteogenic activity reinforces its translational relevance, as these mechanisms are essential for the predictable regeneration of bone defects both in experimental and future clinical contexts.

### 2.5. Bovine Bone

Deproteinized bovine bone is considered a natural hydroxyapatite and is widely recognized as a xenogenic calcium phosphate bone substitute. After removal of organic components, the bone retains its trabecular architecture and porosity, acting as an osteoconductive material [62]. Due to its favorable host response in bone defects, it acts as a support that promotes capillary neoformation, perivascular tissue development, and cell migration in the recipient bed [63]. Clinical studies have demonstrated its effectiveness in bone healing and implant osseointegration during guided bone regeneration [64]. Generally available in blocks and granules (cortical or cancellous), it has a particle size range of 250 to 1000 μm (microgranules) or 1000 to 2000 μm (macro granules). In terms of porosity, it can contain macropores ranging from 300 to 1500 μm, micropores that mimic Haversian canals and small medullary vascular channels, as well as inter-crystalline spaces ranging from 3 nm to 26 nm. These characteristics result in a porosity percentage between 70% and 75%, which favors cell proliferation [65].

A widely used bone substitute in implant dentistry is Bio-Oss^®^ (Geistlich Pharma, Wolhusen, Switzerland), derived from deproteinized and freeze-dried bovine bone, which has a structure like that of human bone marrow, both in morphology and mineral composition [18]. This material is produced through physical sterilization at approximately 300 °C and chemical processes that eliminate proteins, resulting in a porous, natural, and non-antigenic mineral bone matrix [62].

In preclinical models, deproteinized bovine bone has demonstrated excellent space-maintaining properties, slow resorption, and consistent support for new bone deposition, characteristics that explain its wide adoption as a xenogeneic scaffold in both experimental and clinical bone regeneration [18,62,63,64,65].

Collectively, the preclinical findings across these particulate biomaterials demonstrate distinct biological behaviors, degradation kinetics, and mechanical contributions to defect repair. Understanding these differences in animal models provides essential mechanistic insight and serves as the foundation for future translational research. While the evidence summarized here derives from experimental studies, these results guide the development of safer and more effective biomaterials for eventual clinical application in human bone regeneration.

## 3. Fibrin-Based Biomaterials

Fibrin is considered an essential protein for maintaining the natural healing process of the human body. When an injury occurs, fibrinogen present in the blood is converted into fibrin, forming a network that stabilizes the blood clot and serves as a matrix for cell migration and tissue regeneration [66,67].

Due to healing deficiencies in some patients [68], fibrin has been extensively studied by tissue engineering, where it is used, for example, as a scaffold to support cell growth and the formation of new tissues [69,70]. Fibrin scaffolds have advantages such as appropriate host response, biodegradability, and the ability to mimic the extracellular matrix, promoting cell adhesion, proliferation, and differentiation [71]. Within preclinical bone regeneration research, fibrin has gained particular relevance because it can be combined with particulate biomaterials to enhance defect stabilization, support angiogenesis, and improve early osteoid deposition. This combination has been increasingly investigated in animal models as a strategy to optimize osteoconductive and regenerative outcomes [66,69,70].

In addition to these applications, fibrin matrices have also been chemically or structurally modified to improve their mechanical resistance and regulate degradation rates. Approaches such as enzymatic or chemical crosslinking increase fibrin fiber density and slow enzymatic breakdown, while composite scaffolds integrating fibrin with polymers or inorganic phases (such as hydroxyapatite) enhance stiffness, stability, and biological performance [72,73]. These strategies illustrate how fibrin can be tailored to meet different clinical requirements, expanding its applicability in regenerative medicine.

### 3.1. Fibrin Glue

Fibrin glue is widely used in tissue engineering due to its hemostatic, adhesive, and biodegradable properties. It serves as a temporary support for cells, facilitating the regeneration of damaged tissues. Studies have shown that combining fibrin glue with mesenchymal stem cells (MSCs) promotes the regeneration of bone and cartilage defects, with significant improvements in clinical parameters and no postoperative complications [74]. In addition, the application of MSC-derived exosomes in conjunction with fibrin glue has shown potential for improving the morphological and histological properties of the anterior cruciate ligament in rabbit models [75]. However, the effectiveness of fibrin glue may be limited in some cases, leading to the development of more specific and effective alternatives [71]. These limitations have driven the development of more specific and effective alternatives, such as fibrin sealant.

### 3.2. Fibrin Sealant

Fibrin sealant is produced from a combination of an enzyme obtained from rattlesnake venom (*Crotalus durissus terrificus*) and heme-derived substances. It is a natural, biodegradable biopolymer that serves as excellent support for stem cells. Fibrin biopolymers, formerly known as “fibrin glue or sealants”, have hemostatic, adhesive, and sealing properties and are widely used in medical and dental procedures [76].

With properties that promote repair and regeneration, it can act as a scaffold that promotes cell proliferation and neovascularization. In surgical sites, fibrin glue is manufactured artificially and used as a biological adhesive to bond tissues and facilitate healing. One of its main advantages is biocompatibility, which ensures good tolerance by the body, reducing the risk of adverse reactions [76]. In addition, fibrin glue degrades naturally as the tissue heals, eliminating the need for subsequent removal [77].

In the 1990 s, researchers at the Center for the Study of Venoms and Venomous Animals (CEVAP, São Paulo State University, UNESP, Botucatu, Brazil) developed a new heterologous biomaterial. This is prepared from a serine protease extracted from the venom of *Crotalus durissus terrificus* and a cryoprecipitate rich in fibrinogen, obtained from the blood of buffalo (*Bubalus bubalis*). The use of fibrin sealant derived from snake venom, such as that developed by CEVAP, has been successfully applied in experimental research, promoting the application of cells at the site of injury and accelerating the tissue repair process [78,79,80,81].

Its use allows the application of a large number of cells at the site of injury, repair of nerve, skin, and bone tissues, accelerating the tissue repair process and promoting faster patient recovery [81,82]. Studies have also shown that the combination of fibrin sealant with mesenchymal stem cells (MSCs) promoted the regeneration of bone and cartilage defects, showing improvements in clinical parameters and no postoperative complications [74].

Although the terms “fibrin glue” and “fibrin sealant” are sometimes used interchangeably, they refer to formulations with distinct characteristics. Fibrin glue typically contains lower thrombin concentrations, resulting in slower polymerization and allowing deeper tissue penetration, which supports its use as a scaffold in regenerative medicine [71,74,75]. In contrast, fibrin sealants generally contain higher thrombin concentrations and additional components that promote rapid polymerization and strong adhesion, properties that make them suitable as hemostatic and sealing agents in surgical applications [76,77]. These differences explain why fibrin glue is often applied in tissue engineering contexts, whereas fibrin sealants are widely used for hemostasis and wound closure in medical and dental procedures.

### 3.3. Platelet-Rich-Fibrin (PRF)

Fibrin-rich platelets (PRF) are a second-generation platelet concentrate developed from the evolution of Platelet-Rich Plasma (PRP), designated as first generation. Both are obtained by centrifuging the patient’s own blood to concentrate platelets for the release of growth factors that will stimulate healing and tissue regeneration [83,84]. However, PRP has limitations, such as the need for additives such as anticoagulants and external activators (such as thrombin and calcium) that can interfere with the quality of PRP and the release of growth factors [85].

PRF, formulated by Choukroun in 2001 [86], obtained through slower centrifugation of blood, forms a more stable fibrin matrix, requiring no anticoagulants. In addition, it releases growth factors in a gradual and sustained manner, which enhances bone and tissue regeneration, promoting longer-lasting effects and eliminating the risk of adverse reactions associated with the use of additives [87,88]. New discoveries highlight applications in numerous disease treatments in various specialties, such as dermatology, orthopedics, and dentistry, promoting tissue regeneration, early bone healing, and better clinical insertion [84,89,90].

## 4. Association of Biomaterials with Fibrin Derivates

In this review, we analyzed preclinical studies previously published between 2015 and 2025 that report the association of particulate biomaterials with fibrin-derived products for tissue regeneration. The aim is to identify which interventions show promising results in animal and/or in vitro models, providing support for future clinical studies and contributing to the understanding of the possible benefits of their use in humans.

Independent studies from the literature were considered, addressing contexts in clinical and experimental/in vitro applications. The articles analyzed were organized in chronological order in Table 1.

**Figure 2:**
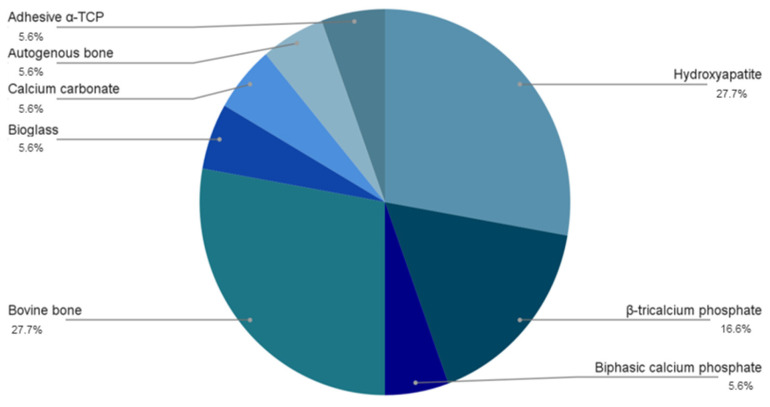
Most frequently used particulate biomaterials in the analyzed studies.

**Figure 3:**
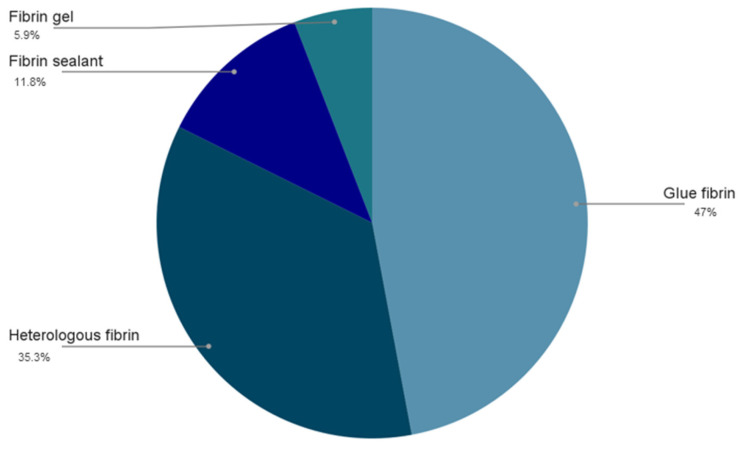
Most frequently used fibrin derivatives in the analyzed studies.

In addition, graphical representations (Figure 2 and Figure 3) were created to facilitate interpretation by the reader, showing the distribution of the prevalence of the different biomaterials used and fibrin derivatives in this study. This approach provides a clear overview of the applications used by the authors for tissue regeneration.

## 5. Comparative Analysis of the Studies Summarized in Table 1

To improve the integration of the concepts discussed in this review, this section provides a structured interpretation of the experimental findings summarized in Table 1. First, the fundamental principles of biomaterials and fibrin-based strategies in bone regeneration are briefly revisited to contextualize their biological relevance. Then, a comparative analysis of the main studies is presented, highlighting consistent trends, divergences, and the translational implications of the evaluated combinations.

Building on the fundamental concepts outlined in the Introduction, the comparative analysis of the studies demonstrates that these biomaterials behave differently depending on their composition and interaction with fibrin derivatives. This variation becomes evident when examining how each material influences bone formation, remodeling dynamics, and the integration of fibrin-based components across preclinical models.

Comparing the experimental studies in Table 1, it was observed that hydroxyapatite (HA) and bovine bone (*n* = 6) were the most widely used particulate biomaterials, followed by β-TCP (*n* = 3) and other biomaterials that were used in only one study, such as bioglass.

It was observed that HA favored organized bone deposition and volumetric persistence, albeit with slow remodeling, a characteristic typical of this biomaterial [91]. β-TCP, on the other hand, promoted more accelerated resorption and replacement by newly formed bone tissue in less time, but with less dimensional stability, which may be a limitation in larger defects [97]. In contrast, the use of bovine bone in conjunction with fibrin biopolymer, especially when associated with complementary therapies such as photobiomodulation, obtained intermediate results [104].

When individual studies are compared directly, additional nuances emerge. For example, Chao et al. [91] and Suloglu et al. [93] both evaluated hydroxyapatites associated with fibrin-based materials, but Suloglu et al. [93] reported greater bone volume and more advanced maturation, likely due to the use of a fibrin sealant rather than fibrin glue. Similarly, Tan et al. [97] and Kageyama et al. [103] investigated β-TCP combined with fibrin derivatives and observed faster remodeling than HA-based constructs, although Kageyama et al. reported superior matrix organization, possibly due to differences in fibrin concentration and defect size. Studies using bovine bone, such as Sadeghinia et al. [95] and Buchaim et al. [101], produced intermediate outcomes, but Buchaim’s model yielded a more homogeneous trabecular pattern, an effect attributed to the concomitant use of photobiomodulation. Finally, the findings of Zhao et al. [99] involving bioglass differed from all other studies due to the material’s high bioactivity and ion-release profile, highlighting its distinct mechanism of action compared with calcium-phosphate scaffolds.

It is important to emphasize that comparisons between particulate biomaterials must be carried out with diligence, since each study used a different experimental protocol, including differences in the type and proportion of materials used and even the combination of adjuvant therapies, such as photobiomodulation. The heterogeneity makes it difficult to standardize results, and comparisons must be made in a generalized manner. This methodological heterogeneity includes variations in sample size, animal species, defect dimensions, and biomaterial proportions, which naturally limit direct comparability across studies.

Given these discrepancies, the consistency of the findings must be interpreted cautiously, since differences in protocols can partially explain the variation in bone formation rates across studies. Overall, although there is no consensus due to methodological differences, the findings suggest that tricalcium phosphate associated with fibrin sealant performed more favorably in terms of bone remodeling and neoformation over the study period [97].

Bioglass, even though used in only one study [99], showed relevant benefits, such as increased bone formation, improved mechanical strength, and sustained release of bioactive ions. These findings reinforce the bioactivity potential of bioglass when combined with fibrin derivatives. However, as this is isolated evidence, further studies are needed to evaluate this combination in different contexts to validate its translational applicability.

Regarding the use of fibrin derivatives, studies indicate that their adhesiveness, in combination with biomaterials, significantly stimulates bone neoformation [107]. Fibrin glue was the most widely used derivative among the studies (n = 8), and despite having important hemostatic and adhesive properties, it has limited translational evidence. Among fibrin sealants, the only fully heterologous one that has been widely documented is the one developed by the Center for the Study of Venomous Animals (CEVAP, UNESP, Botucatu, Brazil), which has demonstrated its effectiveness in six studies [94,98,100,101,104,106]. Conclusive results suggest that the product is safe, biocompatible, and shows promising efficacy.

In contrast, platelet-rich fibrin (PRF) differs from adhesives in that it is autologous and acts primarily as a source of growth factors. It is worth noting that PRF already has clinical applicability, mainly in dentistry, with randomized studies showing its advantages in maxillary sinus lifting, alveolar preservation, and periodontal regeneration [108,109,110]. In this context, PRF is considered a clinical reference, while fibrin glue and sealants still require additional validation in experimental and translational research.

**Figure 4:**
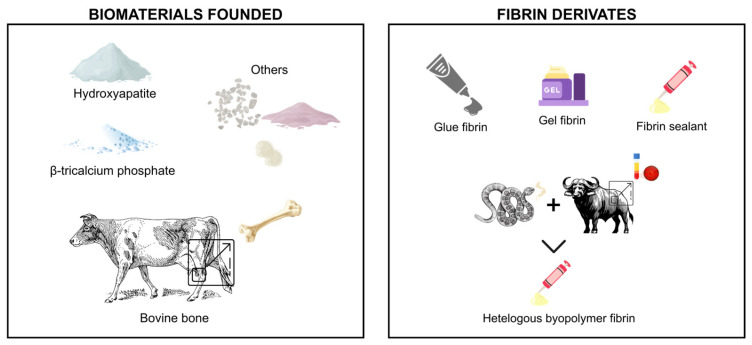
Main particulate biomaterials and fibrin derivatives.

**Figure 5:**
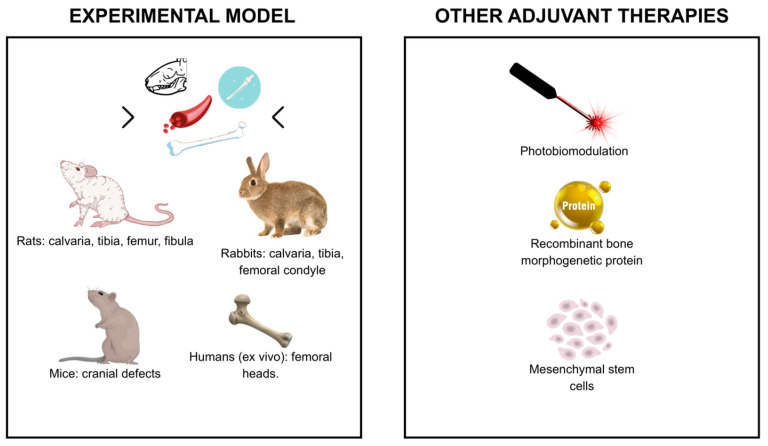
Experimental models and adjuvant therapies.

For a clearer understanding of the trends discussed above, Figure 4 and Figure 5 compile the main particulate biomaterials and fibrin derivatives, as well as the experimental models and adjuvant therapies employed across the included studies.

Therefore, future research should aim to bridge the gap between preclinical findings and controlled clinical trials to validate the efficacy of fibrin sealants in combination with particulate biomaterials. Comparative studies involving different biomaterials, including hydroxyapatite (HA), tricalcium phosphate (TCP), bovine bone, bioglass, and fibrin derivatives (glue, sealant, PRF), are essential to identify the most effective combinations for predictable and consistent bone regeneration.

## 6. Conclusions

In summary, this narrative review examined the preclinical evidence on the combination of particulate biomaterials and fibrin derivatives for bone regeneration, with the aim of identifying recurrent experimental patterns and the biological relevance of these associations. Across the included studies, hydroxyapatite and bovine bone emerged as the most frequently used particulate scaffolds, whereas β-TCP demonstrated faster remodeling but lower volumetric stability. These findings are consistent with the comparative trends summarized in Table 1.

Regarding fibrin-based materials, heterologous fibrin sealants, particularly the CEVAP formulation, showed reproducible favorable host response and regenerative potential in several experimental models, while fibrin glue and platelet-rich fibrin presented distinct roles depending on their adhesive or growth-factor-rich profiles. Altogether, the evidence indicates that combining particulate biomaterials with fibrin derivatives enhances osteo-conduction and supports more organized bone formation under preclinical conditions.

Despite these promising results, the available data remain limited to experimental models, underscoring the need for standardized protocols and well-designed translational and clinical studies to determine the therapeutic applicability of these combinations in human bone repair.

### Future Research Roadmap

Considering current ethical constraints and the growing global effort to reduce, refine, and replace animal use (the 3Rs principle), future investigations should prioritize advanced small-animal models with improved methodological standardization, complemented, when ethically and scientifically justified, by large-animal studies approved by institutional committees. Harmonizing experimental parameters, such as biomaterial particle size, fibrin concentration, and application techniques, will improve the comparability of findings across studies. Furthermore, mechanistic research exploring the molecular and structural interactions between fibrin matrices and the bioactive components of particulate biomaterials may help clarify the synergistic effects observed in bone regeneration. Collectively, these refinements will support the development of reproducible, ethically responsible, and translationally relevant protocols.

## Figures and Tables

**Table 1:** Particulate Biomaterials + Fibrin Derivatives (In Vitro/Experimental).

Study/Year	Type of Biomaterial	Type of Fibrin Derivate	Experimental Model	Outcomes	Limitations
Chao et al. (2015) [91]	Hydroxyapatite (HA) composite microspheres (HA + gelatin)	Fibrin glue (FG)	Rat calvarial defect model (Sprague Dawley rats)	HA microspheres improved bone regeneration vs. FG; high osteo-conductivity	Small sample; short follow-up; no clinical validation
Nam et al. (2017) [92]	Xenogenic bone (CollaOss)	Fibrin glue (FG)	Rat fibula critical-sized defect model	rhBMP-2 + FG enhanced bone formation; early ectopic bone; later remodeling	FG alone minimal effect; limited bone volume long term
Suloglu et al. (2019) [93]	Hydroxyapatite Granules (HA)	Fibrin sealant (bovine)	Rat calvarial critical-sided defect model	HA + FS increased bone and vessel formation vs. controls	Short-term; small model; no mechanical testing
Cassaro et al. (2019) [94]	Biphasic calcium phosphate (BCP: Hydroxyapatite (HA) + β-Tricalcium phosphate (+ β-TCP))	Fibrin biopolymer (snake venom + buffalo fibrinogen)	Rat femoral defect (Wistar rats)	BCP + fibrin biopolymer improved handling, integration, and osteo-conduction	Incomplete bone fill; short duration; rat model
Sadeghinia et al. (2019) [95]	Xenogenic bovine bone mineral (Bio-Oss)	Fibrin glue (FG)	Rat calvarial critical-size defect	Bio-Oss + FG promoted greater bone formation and integration	Partial regeneration; short-term; small model
Gong et al. (2019) [96]	Calcium barbonate (CaCO_3_) microspheres	Fibrin glue hydrogel	Rabbit tibia defect + in vitro nBMSCs	CaCO_3_ + FG hydrogel increased strength, sustained BMP-2 release, faster healing	Complex design; growth factor dependent; preclinical only
Tan et al. (2020) [97]	β-tricalcium phosphate (β-TCP) scaffold	Fibrin Sealant (FS)	Rat calvarial critical-sized defect	β-TCP + FS improved trabeculae thickness and defect filling	Short-term; no clinical data
De Oliveira et al. (2020) [98]	Autologous bone fragments (particulate graft)	Fibrin Sealant (FS) (snake venom + buffalo fibrinogen)	Rat tibial defect (Wistar rats)	FS + autologous bone raised rigidity and mineral density vs. control	Small defect; not load-bearing; limited model
Zhao et al. (2022) [99]	Mesoporous bioactive glass (MBG)	Fibrin glue (FG)	Rat model of rapid maxillary expansion	MBG + FG improved osteogenesis, reduced relapse, and balanced remodeling	Animal model only; no human data
Guastaldi et al. (2022) [100]	Experimental calcium phosphate - ECP (ACP + OCP + HA) compared with DBB and β-TCP	Fibrin sealant (snake venom enzyme + buffalo fibrinogen)	Rabbit calvarial critical-sided defects	ECP + FS increased resorption and bone deposition; comparable to DBB, β-TCP	Animal study only; long-term results unknown
Buchaim et al. (2022) [101]	Xenogenic bone graft (Bio-Oss–bovine hydroxyapatite)	Fibrin sealant (snake venom enzyme + buffalo fibrinogen)	Rat calvarial critical-sized defects + photobiomodulation therapy	Bio-Oss + FS + PBM enhanced bone and collagen deposition	Short follow-up; rat model; limited translation
Bojan et al. (2022) [102]	Phosphoserin e-modified α-tricalcium phosphate (OsStic^®^ bone adhesive)	Fibrin tissue adhesive (Tisseel^TM^) as control	Ex vivo human (osteoporotic bone, hip arthroplasty patients)	OsStic^®^ stronger adhesion vs. FG (123 N vs. 5 N); suitable for fixation	Ex vivo only; no healing evaluation
Kageyamaet et al. (2023) [103]	β-tricalcium phosphate (β-TCP) + collagen microgels with MSCs (bone beads)	Fibrin gel (fibrinogen + thrombin) covering the transplant in rat model	Nude mice (cranial defects) and nude rats (cranial defects)	β-TCP + fibrin gel improved vascularization and bone regeneration	Short-term; small model; scalability uncertain
Pomini et al. (2023) [104]	Deproteinized bovine bone (DBBM) particles	Fibrin sealant (venom snake enzyme + buffalo fibrinogen)	Rat calvarial critical-sized defects + photobiomodulation (PBM)	DBBM + FS + PBM increased bone volume and collagen integration	Incomplete defect repair; experimental proportions
Vigliar et al. (2024) [105]	Hydroxyapatite and collagen composite granules	Autologous fibrin glue (fibrin-based matrix)	Rat cranial critical-sized defects	HA/collagen + fibrin glue accelerated repair and vascularization	Short-term; small animals; no human trials
Rossi et al. (2024) [106]	Nanohydroxyapatite (nHA)	Fibrin sealant (venom snake enzyme + buffalo fibrinogen)	Critical-sized calvarial defect in rats	nHA + FS + PBM enhanced bone regeneration and organization	42-day follow-up only; preclinical model
Liu et al. (2025) [45]	Calf bone granules (bovine bone)	Fibrin glue (used as control)	Rat femoral artery hemorrhage, rabbit femoral condyle defect, rat skull defect	PEG/gelatin hydrogel with bone granules improved retention and healing	≤8 weeks follow-up; no clinical trials

Abbreviations: HA, hydroxyapatite; β-TCP, beta-tricalcium phosphate; BCP, biphasic calcium phosphate; DBBM, deproteinized bovine bone mineral; DBB, deproteinized bovine bone; CaCO_3_, calcium carbonate; MBG, mesoporous bioactive glass; ECP, experimental calcium phosphate; FG, fibrin glue; FS, fibrin sealant; HFB, heterologous fibrin biopolymer; PRF, platelet-rich fibrin; PRP, platelet-rich plasma; MSCs, mesenchymal stem cells; PBM, photobiomodulation; CEVAP, Center for the Study of Venoms and Venomous Animals (UNESP); BMP-2, bone morphogenetic protein-2; rhBMP-2, recombinant human bone morphogenetic protein-2; nHA, nanohydroxyapatite; PHF and PHFL, groups treated with deproteinized bovine bone, fibrin, and photobiomodulation (laser).

## Data Availability

The original contributions presented in the study are included in the article, further inquiries can be directed to the corresponding author.

## Abbreviations

HA	Hydroxyapatite
β-TCP	Beta-Tricalcium Phosphate
BCP	Biphasic Calcium Phosphate
Ca/P	Calcium/Phosphate ratio
CaCO_3_	Calcium Carbonate
ACP	Amorphous Calcium Phosphate
OCP	Octacalcium Phosphate
DBBM	Deproteinized Bovine Bone Mineral
DBB	Deproteinized Bovine Bone
PRF	Platelet-Rich Fibrin
PRP	Platelet-Rich Plasma
FG	Fibrin Glue
FS	Fibrin Sealant
HFB	Heterologous Fibrin Biopolymer
MBG	Mesoporous Bioactive Glass
MSCs	Mesenchymal Stem Cells
PBM	Photobiomodulation
CEVAP	Centro de Estudos de Venenos e Animais Peçonhentos (UNESP)
ECP	Experimental Calcium Phosphate
nHA	Nano-Hydroxyapatite
TG2	Transglutaminase Type 2
EMSCs	Ectomesenchymal Stem Cells
BMP-2/rhBMP-2	Bone Morphogenetic Protein-2/Recombinant Human BMP-2
PHF/PHFL	Groups with Deproteinized Bovine Bone + Fibrin + Photobiomodulation (Laser)
